# Greenspace Inversely Associated with the Risk of Alzheimer’s Disease in the Mid-Atlantic United States

**DOI:** 10.3390/earth2010009

**Published:** 2021-02-28

**Authors:** Jianyong Wu, Laura Jackson

**Affiliations:** 1 Oak Ridge Institute for Science and Education, hosted by US EPA, Office of Research and Development, Research Triangle Park, Durham, NC 27711, USA; 2 US EPA, Office of Research and Development, Center for Public Health and Environmental Assessment, Research Triangle Park, Durham, NC 27711, USA

**Keywords:** greenspace, blue space, Alzheimer’s disease, PM2.5, environmental health

## Abstract

Alzheimer’s disease (AD) is a chronic neurodegenerative disease and the most common form of dementia in older adults. Treatment of AD symptoms is very challenging and expensive. Appropriate diet as well as mental and physical activity may delay or reduce the occurrence of AD. It is unknown whether environmental factors offer potentially protective effects against the development of AD. We explored the possible beneficial effects of greenspace (trees and herbaceous cover) on the rate of AD in the mid-Atlantic US. Data for initial AD medical claims during 2011–2013 were obtained from Medicare records for 2999 ZIP codes. The percentages of land cover classes in each ZIP code were calculated based on high-resolution land cover imagery. Associations between AD and greenspace, blue space (water), and other variables were examined using zero-inflated Poisson models. The rate of AD was negatively associated with greenspace (for a greenspace increase of 10%, risk ratio (RR) = 0.91, 95% confidence interval (CI): 0.89–0.94), and blue space (for a water area increase of 10%, RR = 0.85, 95% CI: 0.81–0.89). The inverse relationships between greenspace and the risk of AD held across season, gender, and race. The rate of AD was positively associated with the concentration of fine particulate matter (PM2.5) (RR = 1.03, 95% CI: 1.02–1.05 for an increase in PM2.5 of 1 μg/m^3^). Our results suggest that greenspace may have protective effects for AD, although potential mechanisms are unclear and require further investigation.

## 1. Introduction

Alzheimer’s disease (AD) is a progressive neurodegenerative disease that gradually impairs memory and thinking skills, leading to the loss of independence and the inability to perform the basic activities of daily life [1]. The overproduction of the amyloid-β (Aβ) peptides and hyperphosphorylation of the Tau protein and its subsequent deposition are hypothesized as two major mechanisms to develop AD [2,3]. While its mechanism is unknown, it is the most common cause of dementia in older adults and is ranked as the sixth leading cause of mortality in the US. More than 5 million people may currently have AD in the US; this number is expected to rise rapidly in the next decades [4,5].

AD is irreversible, and while certain drugs may alleviate some symptoms of AD, none can cure it or stop its progression [6]. Total costs for health care and long-term care are estimated at hundreds of billions of dollars, posing a considerable financial burden on families and making it the most expensive disease in the US [4].

AD may have both genetic and non-genetic factors that play roles in its development [1,7]. Age is the primary risk factor for AD [8] with nearly 95% of AD patients aged 65 and older [1]. Family history is the second largest risk for AD, and genetics may be responsible for 80% of cases [9]. Polymorphisms in ApoE, SORL1, and GSK3 genes are thought to be the major genetic risk factors [10-12]. For non-genetic factors, cerebrovascular disease, high blood pressure, Type 2 diabetes, heavy body weight, high plasma lipid levels, metabolic syndrome, smoking, and traumatic brain injury have been found to be positively associated with AD. Environmental pollutants as a risk factor for AD have also been gradually recognized [10]. Studies have found that metals (mercury, arsenic), insecticides/pesticides, nanoparticles, and air pollutants might induce AD or AD-like progression in animal and human subjects [10,13,14]. Several epidemiological studies have also found that long-term exposure to fine particulate matter (PM2.5), one of the major traffic-related air pollutants, was positively associated with AD incidence [15-17].

Some factors may offer protection from AD. For example, diet (e.g., Mediterranean food), physical exercise, and intellectual activity may reduce AD risk [4,5]. Greenspace, such as trees, gardens, and parks, has been found to provide many human health benefits because of the filtration of air pollutants, promotion of physical activity and social contact, and reduction of stress and depression [18,19]. Recent studies have shown that exposure to greenspace may be beneficial to mental health and brain health [20,21]. Greenspace may be an environmental protective factor for human health, including for AD. However, the relationship between greenspace and AD is unknown. Water views in the landscape have frequently been expressed as a human preference (reflected in real-estate values and vacation destinations) and a restorative element [22,23]. While research on mental health or cognitive benefits of blue space is scarce [24], time spent at the beach has been linked to healthy behavioral development in Barcelona schoolchildren [25]. Therefore, this study examined the association of green and blue spaces with the rate of AD in the mid-Atlantic US. It also sought to replicate previously documented associations between AD rate and PM2.5, and to control for PM2.5 in models since PM2.5 has been identified as a possible risk factor for AD. Furthermore, by assessing natural infrastructure and air pollution in combination, this study may offer insights into possible protective effects of vegetation on AD through particulate entrapment [26].

## 2. Materials and Methods

### 2.1 Study Area

**Figure 1.**
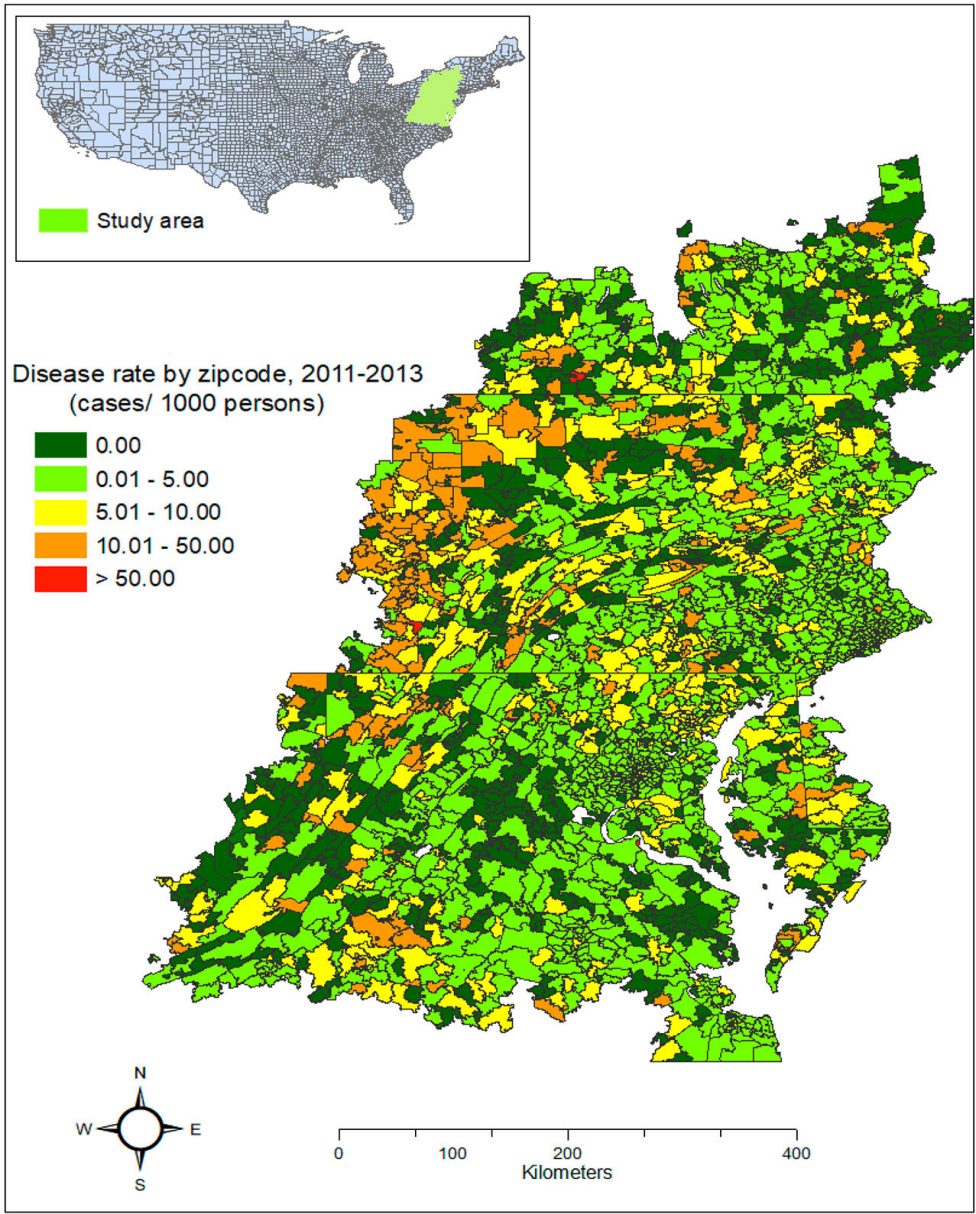
Rate of initial Alzheimer’s disease claims in the study area during 2011–2013.

Our study area encompassed 2999 postal ZIP codes in the mid-Atlantic United States (all or parts of New York, Pennsylvania, Delaware, Maryland, West Virginia, Virginia, and Washington, DC, Figure 1). It was selected because the effects of PM2.5 on human neurological diseases have been observed here [16]. In addition, a high-resolution (1 m) land cover dataset is available for this area. According to the US Census Bureau’s American Community Survey for 2009–2013, the area had a population of 26 million.

### 2.2 Alzheimer’s Disease Data

The AD data were obtained from Medicare enrollees aged ≥ 65 whose medical records are held by the Center for Medicaid and Medicare (CMS). The whole dataset contains the records from 1999 to 2013. In the CMS dataset, each enrollee has a unique identification number and codes to indicate the types of diagnoses according to the International Classification of Disease-Ninth Revision (ICD 9). For enrollees with records of AD (ICD 9 code 331.0) during the study period, we extracted the earliest record for analysis. Specifically, we selected the first record of a patient who was diagnosed with the AD from the dataset and then selected the data for 2011–2013 to most closely match the high-resolution land cover data. Information about the date (year, month, and day) of health care, residential location (ZIP code, county and state), race, and gender were also available. As the specific address for each patient was withheld, we analyzed the data at the ZIP code level. The data were also aggregated by month to correspond to the PM2.5 data.

### 2.3 Land Cover Data

A 2013–2014 classified land cover dataset for the study area was obtained from the Chesapeake Bay Innovation Center [27]. This one-meter resolution dataset is derived from photography collected by the USDA National Aerial Imagery Program and covers approximately 259,000 km^2^ in and around the Chesapeake Bay watershed. The land cover was originally classified into 6 major categories: water, trees (including shrubs), herbaceous, barren, impervious, and roads. We combined the tree and herbaceous classes into one greenspace class and calculated the percentage of each resulting type of land cover by ZIP code using 2010 ZIP code from the US Census Bureau [28].

We also obtained data for major roadways in the study area from NavTEQ™ (Chicago, IL, USA, the leading provid er of maps, traffic and location data in North Americ a). Using; ArcGIS 10.fr (ESRI, CA, USA), we calculated density of mapr road s (interstate, stare highways, and major arterials) by ZIP code using the total length of major roads in a ZIP code divided by the total area of that ZIP code.

### 2.4 PM2.5 Data

We obtained PM2.5 data for the study area from the U.S EPA [29]. These are estimates at the census tract level, which were downscaled from regional models and fused with data from field monitors. We assigned these PM2.5 values to ZIP codes using a nearest neighborhood method. Specifically, we calculated the distances between census tract and ZIP code centroids. A PM2.5 value assigned to a ZIP code was the same as the value of the nearest census tract.

### 2.5 Demographic and Socioeconomic Status Data

We obtained demographic and socioeconomic status data for each ZIP code from the US Census Bureau’s five-year American Community Survey for 2009–2013. We restricted the population data to age 65 and above because the Medicare data represents primarily this age group. Additionally, AD emerges primarily from within this population [1].

We calculated the percentage of this population in each ZIP code by gender and predominant race (white and black) for stratified analyses. We used the median annual household income to indicate socioeconomic status (SES) by ZIP code. Population density was calculated using the total population in a ZIP code divided by its area.

### 2.6 Statistical Analysis

We used the zero-inflated Poisson model [30] to examine the association between AD and exploratory variables since the response variable, the number of earliest-identified AD records in each ZIP code during the study period, is count data with excess zeros. The exploratory variables included monthly average PM2.5 concentration, percent greenspace, percent water area, median annual household income, ZIP code area, population density, and road density. The natural-log transformed population (age ≥ 65) data was used as the off-set term in the model. We checked for outliers in the response variable and removed extreme large values based on the histogram of the data (nearly 1% of observations) from the dataset. Then, we examined multicollinearity among exploratory variables using correlation analysis and variance inflation factors (VIF) [31]. If two or more variables were highly correlated (i.e., *r* > 0.6), only one variable was included in the model. We also selected the exploratory variables based on the value of the Akaike information criterion (AIC). A smaller AIC suggests a model with a better fit. The statistical analysis was conducted with SAS 9.4 (SAS Institute, Inc, Cary, NC, USA).

We used risk ratios (RRs) to assess the strength of associations between AD and the exploratory variables. If an RR was above 1.00, a positive association was assumed, while if it was below 1.00, a negative or inverse association was assumed. We chose the significance level at 0.05. Greenspace and water were modeled in 10% increments to reflect more meaningful land cover change, as the effects of 1% changes in greenspace and water are trivial [21]. Thus, the RRs for greenspace and water indicate the changes in the risk of AD when these land cover variables increase by 10%. To evaluate seasonal effects on the associations, we stratified the monthly data into spring (March, April, and May), summer (June, July, and August), autumn (September, October, and November) and winter (December, January, and February), and then ran the model for each season. We also stratified the model by gender and race.

## 3. Results

### 3.1 Description of AD Data and Explanatory Variables

Monthly AD by ZIP code ranged from 0 to 5, with a mean value of 0.067 and a standard deviation of 0.29 (Table 1). Monthly average PM2.5 concentration by ZIP code was 9.073 ± 2.252 μg/m^3^ (Table 1). Greenspace was the major land cover type, accounting for 85.01 ± 17.51%. Water area accounted for 3.74 ± 9.25% (Table 1). The median annual household income was $29,315 ± 11,805 and the mean population density was 5.3 ± 18.96 persons/km^2^ (Table 1). The percentages of males and females were approximately equal (Table 1). In the study area, the major race was white, accounting for 85.63% of the population. The black population accounted for 9.34%.

The results of Pearson correlation analysis showed that both monthly AD and AD rate were positively correlated with PM2.5, median income, population density, percentages of female and black populations, road density, and ZIP code area (*r* > 0.0, *p* < 0.01), but they were negatively correlated with the percentage of greenspace and water area, and the percentages of male and white populations (Table 2).

### 3.2 Modeled Associations

The results from the final zero-inflated Poisson model are presented in Table 3. Five explanatory variables were included in the final model, which are PM2.5 concentration, the percentage of greenspace, the percentage of water, median income, and population density. The results showed that AD rate was positively associated with PM2.5 (for a 1 μg/m^3^ increase in PM2.5 concentration, RR = 1.03, 95% confidence interval (CI) = 1.02–1.05) (Table 3). In contrast, AD rate had a negative association with greenspace (RR = 0.91, 95% CI = 0.89–0.94) and with water area (RR = 0.85, 95% CI = 0.81–0.89) (Table 3). Both median annual household income and population density had significant negative associations with AD rate (RR = 0.90 and 0.91, respectively) (Table 3).

### 3.3 Seasonal Effects on the Associations

**Figure 2.**
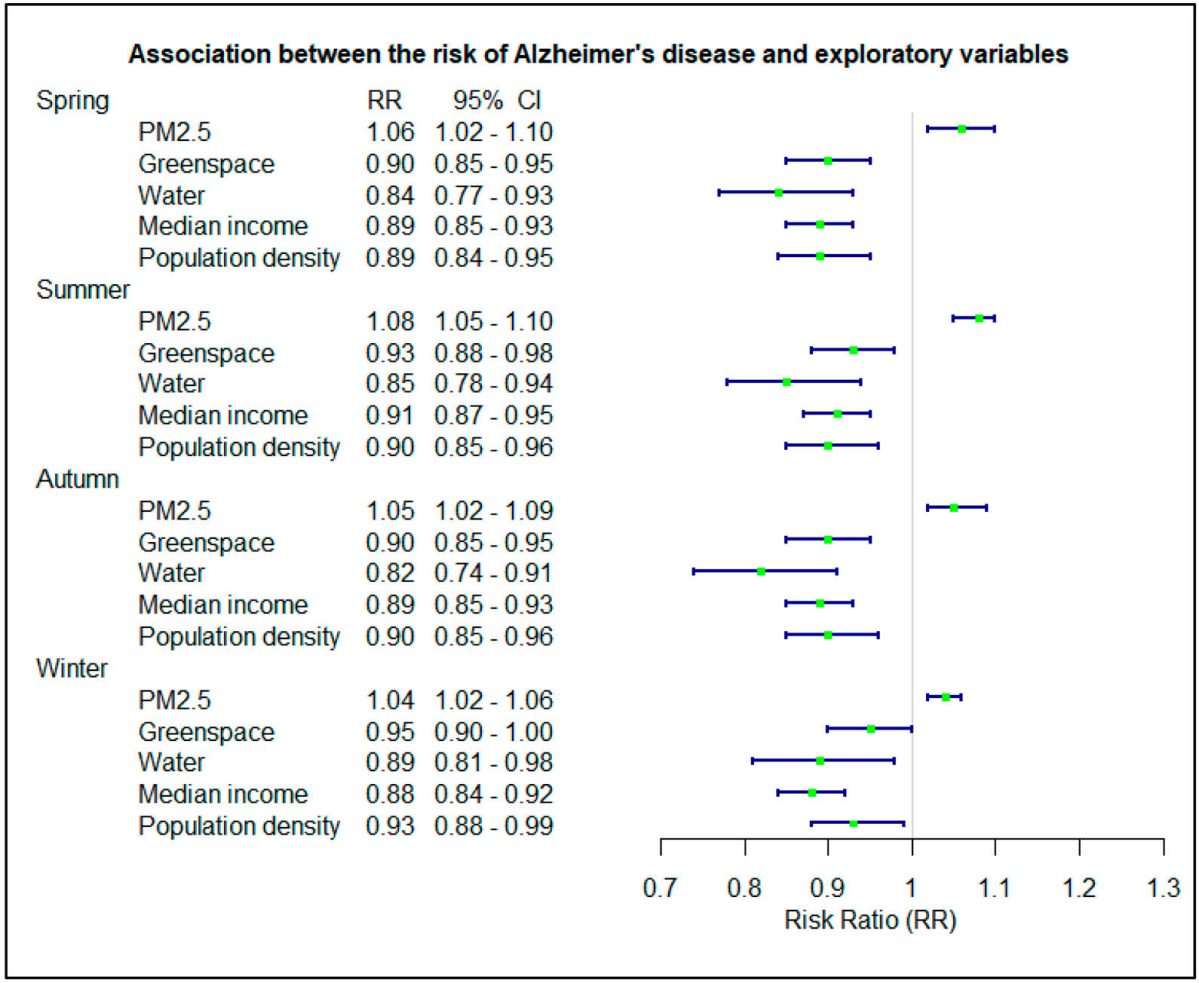
Association between the risk of Alzheimer’s disease and exploratory variables in different seasons.

AD was positively associated with PM2.5 across seasons. The association was slightly stronger in the summer (RR = 1.08, 95% CI = 1.05–1.10) and weaker in the winter (RR = 1.04, 95% CI = 1.02–1.06) (Figure 2). Negative associations were observed for greenspace, water, median income, and population density for all four seasons (Figure 2). The association with greenspace was slightly stronger in spring and autumn but slightly weaker in winter. Similarly, the association with water was slightly stronger in autumn but slightly weaker in winter. Overall, the associations in winter were slightly weaker than those in other seasons, but the differences were not statistically significant (Figure 2).

### 3.4 Association Stratified by Gender and Race

**Figure 3.**
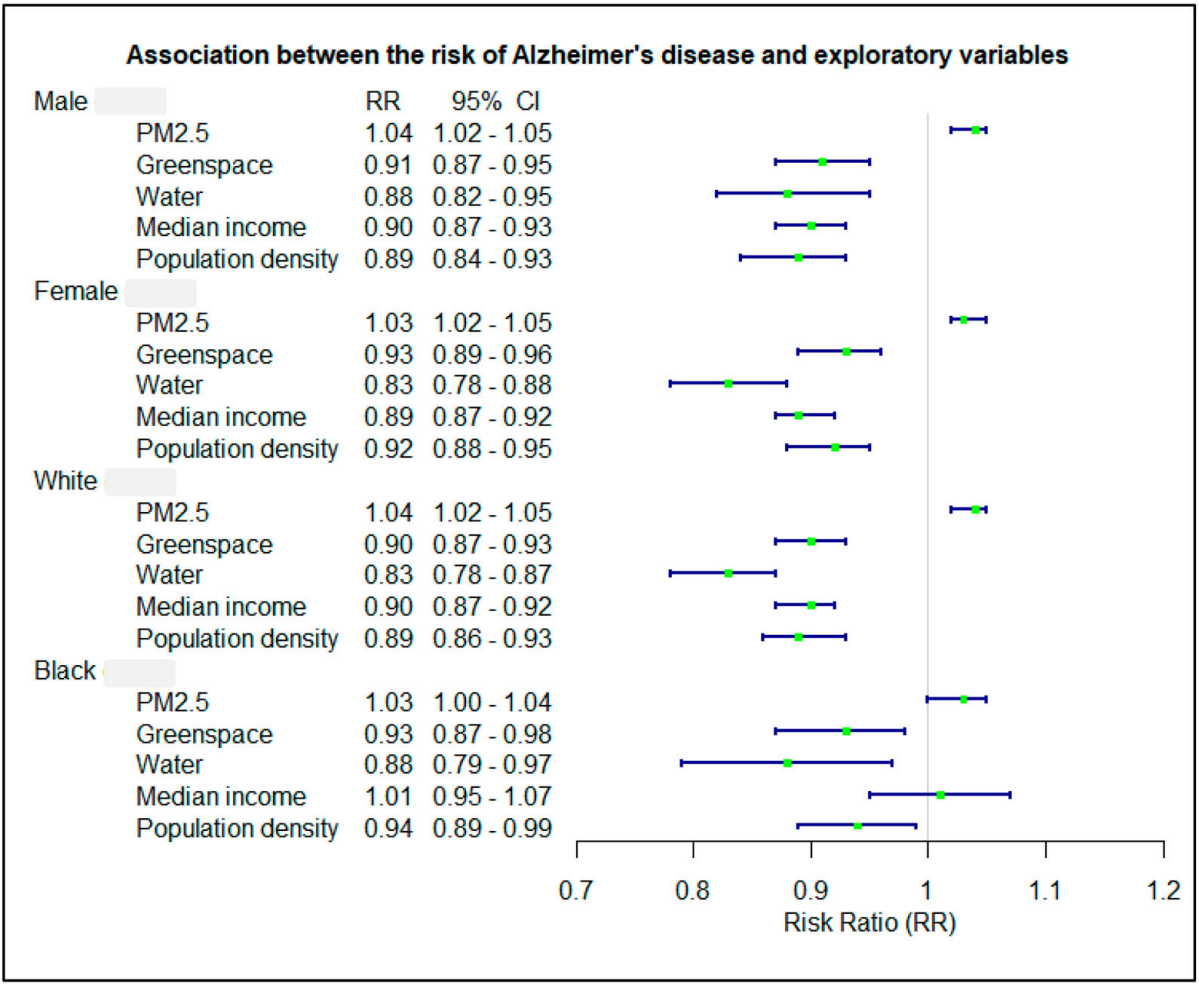
Association between the risk of Alzheimer’s disease and exploratory variables in different gender and race.

The gender and race stratified models showed that associations between AD rate and exploratory variables were similar across strata (Figure 3). However, the association with PM2.5 was extremely significant (*p* < 0.01) in white subjects but only slightly significant (*p* = 0.05) in black subjects. The AD rate had a significant negative association with median income for white patients but not for black patients (Figure 3).

## 4. Discussion

We conducted a large-scale ecological analysis to explore the connection between AD rate and exposure to greenspace and blue space in the mid-Atlantic US. By analyzing AD from Medicare records and land cover measured by high-resolution imagery, we found a significant inverse relationship between AD rate and the percentages of greenspace and water area. The results were consistent when we stratified the model by season, gender, and race and controlled for confounding variables including income and population density. Our study is the first to investigate the possible protective effects of greenspace and blue space on AD rate. Findings from this study suggest that exposure to greenspace and blue space may reduce the risk of developing or delay the onset of AD, providing a new insight to mitigate the high incidence of the disease.

The negative association between greenspace and AD rate in the study area may be due to air pollutant filtration by frees and other vegetation [32,33]. It was estimated that nearly 17,400 million kg of air pollutants were removed by forests and trees in the conterminous United States in 2010 [34]. Herbaceous cover has also been found to take up air pollutants, including PM2.5 [35]. Previous studies have shown that air pollutants are one of the major environmental risk factors for AD, especially traffic-related air pollutants such as PM2.5 [15-17]. Our previous study also suggested that greenspace may have a benefit to brain health through buffering traffic-related air pollution [21]. Generally, air pollution is more serious in urban than in rural areas. However, rural areas can have elevated vehicular air pollutant levels due to diesel highway trucks and farm vehicles.

Greenspace may also reduce the risk of AD by promoting physical activity such as jogging, walking, and biking. Physical exercise may support the maintenance of brain volume, and mitigate obesity, hypertension, stroke, and other AD risk factors [36]. Physical activity has been negatively associated with dementia and is generally regarded as protective [37]. Greenspace (e.g., greenway trails, parks, and gardens) provides attractive and safe places for physical activities, and as such, it may confer substantial health benefits [38-40]. Furthermore, greenspace may play a protective role in the risk of AD through depression reduction. The connection between depression and AD has long been recognized [41,42]. For example, one study has shown that depressed patients were more cognitively impaired and more disabled in daily activities [42]. A systematic review and meta-analysis concluded that late-life depression has positive associations with the risk for Alzheimer’s disease and all-cause dementia [43]. Exposure to tree canopy, which is best associated with perceived greenspace [44], may reduce the risk of dementia such as AD through stress reduction. A large study in Australia showed exposure to tree canopy was associated with a lower risk of dementia [45]. Meanwhile, an inverse association between neighborhood greenspace and depression has been observed in several studies [46-48], suggesting that greenspace may improve neurological health. Greenspace also provides a setting for social interaction [49,50] and engagement with nature, both of which have been associated with mood [51,52] and are beneficial particularly for the elderly [53].

Similarly, we observed a negative association between water and AD rate. The beneficial effects of proximity to blue space may also be related to the promotion of physical activities and social interaction, and stress reduction [22,24,53]. Relative to PM2.5, water does not filter air pollutants directly; however, surrounding wetlands may decrease concentrations of air pollutants, including particulate matter [54]. In addition, pollutants and particulates are more likely to be suspended in the air when it is very dry; near water areas with higher humidity, air pollutants may be reduced [55].

Our results also revealed that the risk of AD was negatively associated with household median income and population density. It is possible that the causal mechanisms involved in AD differ in urban versus rural occupations, and that air filtration, physical activity, and other benefits of greenspace vary across urban and rural landscapes and vegetation types.

One major strength of this epidemiological analysis is that we used high-resolution classified land cover to measure greenspace. Many previous studies used the normalized difference vegetation index (NDVI) to quantify average “greenness” or used medium-resolution remotely sensed images to calculate greenspace. The 1-m land cover data provide more accurate measures of greenspace than those in other studies. Second, our study was conducted across a large spatial extent. Since factors associated with AD are expected to be subtle, the larger spatial extent of the data may allow better discrimination of potential associations, thus potentially increasing the significance of the association between greenspace and the risk of AD.

Our study has a few limitations. First, we do not have any information about where, when, or how long residents were exposed to greenspace. Therefore, the exposure is unclear and is represented only by the percentage of greenspace in the residential Zip code. Second, because of the scarcity of high-resolution imagery, we investigated only three-year data, which is a relatively short period for studying AD. Furthermore, our study was observational rather than experimental, and it was conducted at the ZIP code level instead of the individual level due to the shortage of finer spatial resolution of the Medicare data. The associations at the ZIP code level are more likely subject to confounding bias if the background rate of the disease is correlated with those confounding factors. This study design cannot confirm a causal relationship between greenspace or blue space and AD rate. Although this ecological study has many limitations due to data availability and the difficulty of the question, this work is the first to explore potential health benefits of green space on AD. Given the high rate of AD, exposure to greenspace and blue space may be a feasible way to delay or mitigate the development of AD, thus reducing the cost of AD health care and the suffering of AD patients and their families.

## 5. Conclusions

We observed a lower AD rate associated with increasing greenspace and water area in the mid-Atlantic US. This relationship remained when our model was adjusted for PM2.5, income, and population density, and it was consistent across seasons, gender, and race. The possible benefits of greenspace and blue space may occur through multiple pathways, but as of yet, these are unclear and require further investigation.

## Figures and Tables

**Table 1.** Descriptive statistics of key variables by ZIP code.

Variables	No. Observations	Mean	Standard Deviation	Minimum	Maximum
Medicare claims data					
Initial AD claims/month	106,763	0.067	0.290	0	5
Monthly claim rate	106,763	2.6 × 10^−6^	1.3 × 10^−6^	0	3.98 × 10^3^
Environmental data					
PM2.5 μg/m^3^)	106,763	9.073	2.252	4.485	0.053
Greenspace (%)	106,763	85.013	17.508	7.08	99.77
Water (%)	106,763	3.735	9.248	0	85.73
Covariates					
Median income ($)	105,073	29315	11805	2542	135865
Male population (%)	106,763	50.028	7.753	0	100
Female population (%)	106,763	49.972	7.753	0	100
White population (%)	106,763	85.623	19.828	0	100
Black population (%)	106,763	9.338	16.976	0	100
ZIP code area (km^2^)	106,763	80.564	100.716	0.007	872.515
Population density (1000/km^2^)	106,763	5.300	18.961	0.001	712.326
Road density (km/km^2^)	109,405	0.789	1.711	0	58.555

Medicare claim: An application for Medicare coverage of a medical visit or procedure. Monthly rate: monthly initial AD records/population aged 65 and above.

**Table 2.** Pearson correlation between monthly initial records and rates of Alzheimer’s disease and exploratory variables.

Variables (Unit)	Monthly Initial Records		Monthly Rate	
	*r*	*p*	*r*	*p*
PM2.5 μg/m^3^)	0.069	<0.001	0.050	<0.001
Greenspace (%)	−0.099	<0.001	−0.057	<0.001
Water (%)	−0.002	<0.001	−0.021	<0.001
Median income ($)	0.030	<0.001	0.027	<0.001
Population density	0.059	<0.001	0.060	<0.001
Male population (%)	−0.047	<0.001	−0.026	<0.001
Female population (%)	0.0465	<0.001	0.026	<0.001
White population (%)	−0.125	<0.001	−0.076	<0.001
Black population (%)	0.104	<0.001	0.061	<0.001
Road density (km/km^2^)	0.031	<0.001	0.025	<0.001
ZIP code area (km^2^)	0.062	<0.001	0.062	<0.001

**Table 3.** Association between Alzheimer’s disease and exploratory variables.

Exploratory Variables (Unit)	RR	95% CI	*p*
PM2.5 (μg/m^3^)	1.03	1.02–1.05	<0.001
Greenspace (10%)	0.91	0.89–0.94	<0.001
Water (10%)	0.85	0.81–0.89	<0.001
Median income ($10,000)	0.90	0.88–0.92	<0.001
Population density (1000/km^2^)	0.91	0.88–0.93	<0.001

## Data Availability

The data will be deposited in ScienceHub at EPA except the Alzheimer’s disease data, which are held by the Center for Medicaid and Medicare (CMS).
